# Insertional mutagenesis using the Sleeping Beauty transposon system identifies drivers of erythroleukemia in mice

**DOI:** 10.1038/s41598-019-41805-x

**Published:** 2019-04-02

**Authors:** Keith R. Loeb, Bridget T. Hughes, Brian M. Fissel, Nyka J. Osteen, Sue E. Knoblaugh, Jonathan E. Grim, Luke J. Drury, Aaron Sarver, Adam J. Dupuy, Bruce E. Clurman

**Affiliations:** 1 Divisions of Clinical Research, Fred Hutchinson Cancer Research Center, Seattle, WA 98109 USA; 2 Public Health Sciences, Fred Hutchinson Cancer Research Center, Seattle, WA 98109 USA; 3 Human Biology, Fred Hutchinson Cancer Research Center, Seattle, WA 98109 USA; 4 Present Address: University of Utah, Salt Lake City, UT USA; 5 Present Address: Boston University School of Medicine, Boston, MA USA; 6 Department of Veterinary Biosciences, College of Veterinary Medicine, The Ohio State University, Columbus, OH 43210 USA; 7 VA Puget Sound Health Care System, Seattle, WA 98108 USA; 8 Department of Anatomy & Cell Biology, University of Iowa, Iowa City, IA 52242 USA; 9 Institute for Health Informatics, University of Minnesota, Minneapolis, MN 55455 USA

## Abstract

Insertional mutagenesis is a powerful means of identifying cancer drivers in animal models. We used the Sleeping Beauty (SB) transposon/transposase system to identify activated oncogenes in hematologic cancers in wild-type mice and mice that express a stabilized cyclin E protein (termed cyclin ET74AT393A). Cyclin E governs cell division and is misregulated in human cancers. Cyclin ET74AT393A mice develop ineffective erythropoiesis that resembles early-stage human myelodysplastic syndrome, and we sought to identify oncogenes that might cooperate with cyclin E hyperactivity in leukemogenesis. SB activation in hematopoietic precursors caused T-cell leukemia/lymphomas (T-ALL) and pure red blood cell erythroleukemias (EL). Analysis of >12,000 SB integration sites revealed markedly different oncogene activations in EL and T-ALL: *Notch1* and *Ikaros* were most common in T-ALL, whereas ETS transcription factors (*Erg* and *Ets1*) were targeted in most ELs. Cyclin E status did not impact leukemogenesis or oncogene activations. Whereas most SB insertions were lost during culture of EL cell lines, *Erg* insertions were retained, indicating Erg’s key role in these neoplasms. Surprisingly, cyclin ET74AT393A conferred growth factor independence and altered Erg-dependent differentiation in EL cell lines. These studies provide new molecular insights into erythroid leukemia and suggest potential therapeutic targets for human leukemia.

## Introduction

Insertional mutagenesis is a powerful means of identifying the molecular drivers of cancer initiation and progression in animal models. Sleeping Beauty (SB) is a transposon/transposase insertional mutagenesis system that is designed to either overexpress nearby genes or inactivate genes, depending on the transposon’s integration site and orientation^1,2^. By combining conditional expression of the SB transposase with the T2Onc transposon in various genetic backgrounds, SB screens have been used extensively to identify\cancer genes and how they cooperate with one another in wild type and cancer-sensitizing backgrounds, and across many cancer types^3–6^.

In this study, we employed SB to identify oncogenes that might promote multi-step carcinogenesis in a mouse model engineered to express a stabilized version of the cyclin E protein. Cyclin E, in conjunction with its catalytic partner CDK2, has crucial roles in cell division, and cyclin E-CDK2 deregulation causes genome instability and contributes to cancer development and progression^7^. One important means of cyclin E regulation is phosphorylation-dependent degradation by the SCF^Fbw7^ ubiquitin ligase^8–12^. To study the physiologic consequences of abnormal cyclin E degradation, we previously created a knock-in mouse model that ablated two cyclin E phosphorylation sites (T74 and T393) that trigger its degradation^13,14^. The cyclin ET74AT393A mutation caused increased cyclin E abundance and epithelial cell hyperproliferation. However, these mice did not spontaneously develop epithelial dysplasia or tumors, suggesting that compensatory mechanisms maintain tissue architecture and suppressed tumorigenesis. Cyclin ET74AT393A expression also caused ineffective erythropoiesis with marked expansion of immature erythroid precursors in the spleen and bone marrow, impaired erythroid differentiation, and mild anemia. These features resemble the early stages of human refractory anemia/myelodysplastic syndrome (MDS).

Because MDS can evolve to leukemia in humans, we speculated that cyclin ET74AT393A mice may provide a sensitized background to identify genetic events that cooperate with abnormal cyclin E regulation to promote leukemia. We thus used interferon-inducible Mx-Cre to activate the SB transposase in hematopoietic precursors to identify genes that might cooperate with abnormal cyclin E regulation to promote leukemia. The stabilized cyclin E allele neither predisposed mice to hematologic cancers nor altered gene activations by SB. Strikingly however, Mx-Cre-induced SB activation caused highly penetrant hematologic cancers within 8–13 weeks after Cre induction. To control for biases in transposon integrations that frequently occur proximal to the T2Onc array^15,16^, we used two different T2/Onc2 strains that contained the transposon array on different chromosomes. The most common malignancies were immature T-cell leukemia/lymphomas (T-ALL) and pure red blood cell erythroleukemias (EL), and there was a non-significant trend towards more EL in the cyclin ET74AT393A mice.

To identify activated oncogenes in these neoplasms, we determined the transposon insertion sites in all ELs and T-ALLs. Transposon insertions that are shared by multiple independent tumors, termed common insertion sites (CIS), often occur in the vicinity of cancer-associated genes, which provides the selective pressure for these shared insertions. We identified CIS using two different statistical methods and found that the CIS profile of ELs and T-ALLs differed markedly. Whereas Notch and Ikaros insertions were most common in T-ALL, ETS family transcription factors (*Erg* and *Ets1*) were the most commonly activated genes in ELs and were activated in the almost all of these tumors.

While T-ALL is common in SB screens performed in blood cells, EL has not, and we thus examined EL in more detail by developing 5 transplantable EL cell lines. Transposon analyses indicated that the vast majority of CISs found in primary ELs were lost during culture of EL cell lines, suggesting that they were not required for their proliferation and maintenance *in vitro*. However, all EL lines retained their *Erg* insertions and overexpressed ERG protein, further supporting the key role of *Erg* activation in EL. Insertions near the Bach2 transcription factor were also retained in several cell lines, and one EL line also retained a *Flt3* insertion and exhibited FLT3-dependence. Finally, although cyclin ET74AT393A expression did not impact leukemogenesis or CIS involvement, we found two phenotypic differences between EL cell lines derived from WT and cyclin ET74AT393A mice. First, while WT EL lines remained dependent on exogenous growth factors (GFs), the cyclin E mutant cell lines did not and proliferated in the absence of GFs. Next, *Erg* knockdown resulted in erythroid differentiation of the WT but not the cyclin ET74AT380A cell lines. These findings suggest that properly regulated cyclin E activity influences both growth factor requirements and differentiation in EL cells.

## Results

### Development and Characterization of SB-induced hematologic cancers

**Figure 1:**
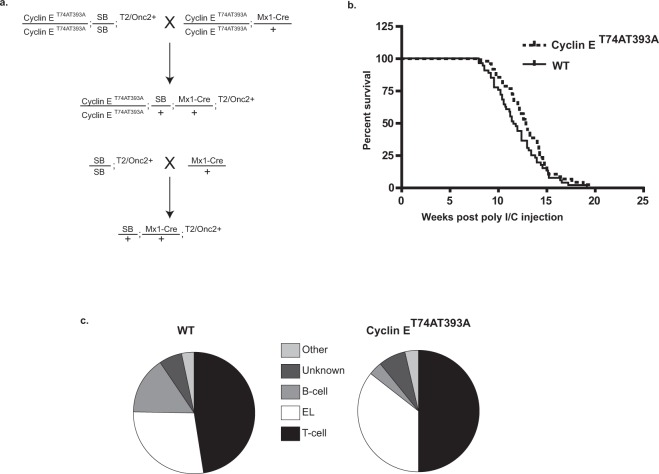
Breeding scheme, survival, and tumor spectrum of Mx-1 sleeping beauty mice. (**a**) Breeding scheme for transposon mutagenesis in cyclin ET74AT393A and wild-type mice. Cyclin ET74AT393A or wild-type mice homozygous for both a T2/Onc transposon array and Cre recombinase-inducible SB transposase allele (T2/Onc2/T2/Onc2; RosaSBLSL/RosaSBLSL) were mated with cyclin ET74AT393A mice heterozygous for Mx-1 Cre to generate cyclin ET74AT393A or wild-type mice with T2/Onc transposon array and Mx-1 Cre recombinase. (**b**) Kaplan Myer survival curve of SB-Cyclin ET74AT393A and SB-wild-type mice following induction of Mx-1 Cre and transpose expression by poly I/C injection. Time indicates intervention due to illness related to hematologic malignancy. Induction of malignancy was completely penetrant in both backgrounds with a median survival of 11.9 weeks post-injection for wild-type and 12.9 weeks for cyclin ET74AT393A mice (p = 0.08). (**c**) Spectrum of hematologic neoplasms that arise in Mx-1 Sleeping beauty mice. Diagnosis was made by a combination of histology and flow cytometry (see text).

We performed a SB insertional mutagenesis screen in WT and cyclin ET74AT393A mice. Mice homozygous for the cyclin ET74AT393A mutation and Rosa26SB11 LSL SB transposase and containing one of two different T2/Onc2 arrays were crossed with mice homozygous for cyclin ET74AT393A and expressing the Mx1-Cre transgene, which generated the experimental cohort (cyclin ET74AT393A; Rosa26SBLSL; T2/Onc2+; Mx1-Cre1) (Fig. 1a). The matched control cohort was wild-type cyclin E; Rosa26SBLSL; T2/Onc2+; Mx1-Cre1. Parallel crosses were made using two separate T2/Onc2 lines with transposon arrays on different chromosomes (strain 6113- chromosome 1, strain 6070 -chromosome 4)^17,18^ Mice (7–10 weeks old) were injected with poly I:C to activate Cre recombinase expression and subsequent transposase expression. Mx1-Cre induction and SB expression was monitored by loss of GFP expression in peripheral blood leukocytes from a subset of study mice, which demonstrated 71–86% loss of GFP-expressing cells following induction (Table S1).

**Figure 2:**
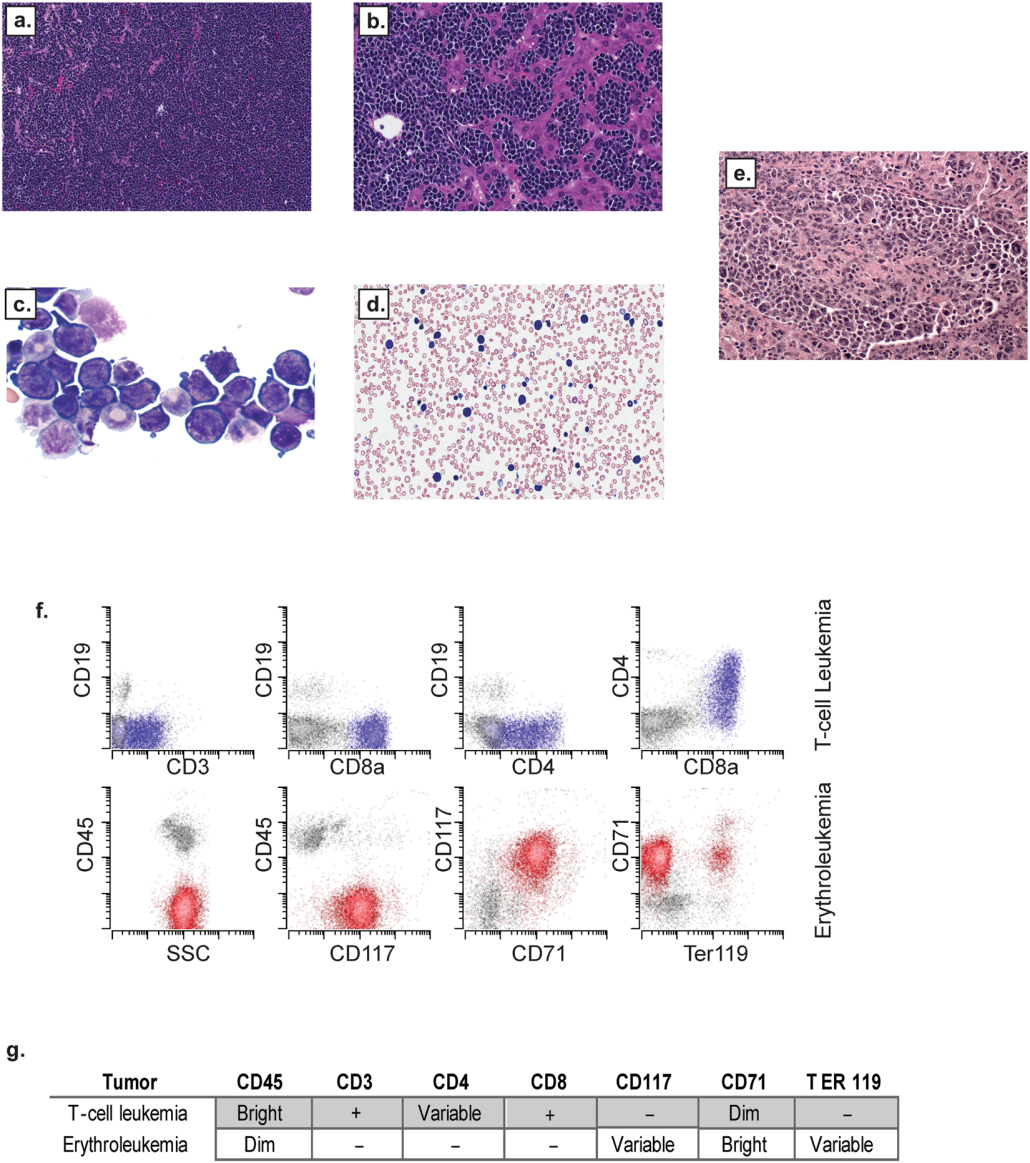
Morphology and immunophenotype of hematopoietic neoplasms in Mx-1 sleeping beauty mice. (**a**,**b**) Histologic sections (H&E) of erythroleukemia involving spleen (A: 20X objective) and liver (B: 40X objective). (**c**) Bone marrow cytospin preparations (Wright Giemsa) of erythroleukemia with block-like clumped chromatin and dark blue basophilic cytoplasm characteristic of erythroid precursor cells (63X objective). (**d**) Peripheral blood smear (Wright Giemsa) with circulating erythroblasts (40X objective). (**e**) Histologic section (H&E) of erythroleukemia with prominent megakaryocytic differentiation (large atypical cells) involving the liver (20X objective). (**f**) Representative immunophenotype of immature T-cell leukemia in blue (top row) and erythroleukemia in red (bottom row). (**g**) Summary of T-cell leukemia and erythroleukemia immunophenotype.

SB insertional mutagenesis in immature hematopoietic cells resulted in progressive serious illness, including weight loss, poor grooming, and hunched posture, leading to euthanasia within 3 months of Mx-Cre induction. Necropsy showed hematologic malignancies in all animals, including lymphoid, myeloid, erythroid and megakaryocytic tumors (Fig. 1c, Table S2). Surprisingly, rather than accelerating tumorigenesis, the cyclin ET74AT393A allele imparted a non-significant trend towards longer survival (11.6 weeks post injection for wild-type and 12.9 weeks for cyclin ET74T393A mice, p = 0.08) (Fig. 1b). The most frequent neoplasm was immature T-cell lymphoblastic lymphoma with leukemia (T-ALL), which usually presented with a massively enlarged thymus. T-ALL typically involved the spleen and bone marrow and in some instances, disseminated to the liver, kidneys and lungs. Leukemia was also noted with leukocytosis and circulating lymphoblasts. Histologic sections of affected organs revealed homogeneous tumors composed of tightly packed lymphoblasts with a high mitotic rate and scattered apoptotic cells admixed with phagocytic histiocytes (Fig. S1). Immunophenotyping by flow cytometry showed expression of CD3 and CD8 with frequent variable co-expression of CD4, findings consistent with immature T-cell lymphoblastic leukemia **(**Fig. 2f,g, Fig. S1, Table S2).

Many (40%, n = 100) mice developed EL with intermixed megakaryocytic differentiation, which caused prominent splenomegaly and involved the bone marrow and sometimes the liver (Figs 1c, 2a,b,e and Table S2). The mice with EL exhibited peripheral involvement with leukocytosis consisting of a prominent population of circulating erythroblasts with dark basophilic cytoplasm (Fig. 2c,d). Multiparameter flow cytometry studies revealed immature erythroblasts (as shown by CD45/side scatter) with dim CD45 expression, variable CD117 (c-Kit) expression, bright CD71 (transferrin receptor) expression, and occasional Ter119 (glycophorin associated protein) expression (Fig. 2f,g, Table S2). ELs varied somewhat with respect to their degree of erythroid differentiation, with variable expression of CD117 (immature immunophenotype) and Ter119 (mature immunophenotype) (Fig. S2). The immunophenotype of the neoplastic population was identical in samples isolated from peripheral blood, spleen and bone marrow (Fig. S3). Some ELs showed megakaryocytic differentiation with scattered enlarged cells with abundant cytoplasm and multilobulated nuclei, morphologically consistent with megakaryocytes (Fig. 2e). Additional flow cytometry studies showed variable expression of CD41, consistent with megakaryocytic differentiation in these cases (Fig. S4). Overall, these tumors displayed many features resembling those of pure red blood cell EL in humans (see Discussion)^19^. Some mice (7%) contained mixed tumors of both lymphoid and erythroid lineages identified by parallel flow cytometry studies (Table S2). The spectrum of hematologic malignancies was similar in the cyclin E-T74AT393A and wild-type mice, with a small trend toward increased EL in the mutant cyclin E mice (Fig. 1c).

### Common Insertion Site (CIS) Identification in T-ALL and EL

To identify CIS, we isolated DNA from 40 ELs (spleens) and 53 T-ALLs (thymus or lymph node), amplified the transposon integration sites via ligation-mediated PCR, and identified the transposon integration sites by next generation sequencing. We mapped 5002 EL insertion sites and 7012 T-ALL sites (Table S3). Each tumor contained between 50 and 150 unique insertions, presumably representing both driver and passenger insertions. We identified CIS by using two different statistical methods that differ in the parameters used to identify CIS and their associated genes: (1) gene-centric common insertion site analysis (gCIS), and (2) TAPDANCE^20,21^.

gCIS identified 74 total CIS in T-ALLs (Table S4). The chromosome that contains the transposon concatemer (the “donor chromosome”) is disproportionately targeted by transposon insertions (“local hopping”) in SB screens. As expected, many CIS were mapped to the donor chromosome. Although these insertions could represent true genetic drivers of cancer, we initially filtered these CIS from subsequent analyses, resulting in 25 T-ALL CIS (Table 1). This analysis identified many established drivers of T-ALL, including *Notch1, Ikzf1, Rasgrp1, and Akt2*^6^. The presence of the cyclin E mutation did not impact the spectrum of CIS observed. Since T-ALLs have been well studied in insertional mutagenesis studies, we focused on the ELs for the remainder of our study^4,22–24^.

**Table 1:** T-cell Lymphoma/Leukemia GCIS (53 tumors total).

Gene Symbol	# Tumors	Chromosome
*Notch1*	40	2
*Ikzf1*	28	11
*Erg*	21	16
*Rasgrp1*	19	2
*Akt2*	16	7
*Runx1*	10	16
*Zmiz1*	9	14
*Foxp1*	8	6
*Akt1*	7	12
*Zbtb42*	7	12
*Kras*	6	6
*Sfi1*	5	11
*Stat5b*	5	11
chr2:98502863-98506672	5	2
*Nedd9*	4	13
*Ptger4, Ttc33*	4	15
*Crebbp*	4	16
chr17:33611661-33655856	4	17
*Scai*	4	2
*Sik3*	4	9
*Pik3r5*	3	11
*Nfil3*	3	13
*Sos1*	3	17
*Itpr1*	3	6
*Myo16*	3	8

gCIS identified 36 CIS genes in ELs prior to filtering out the donor chromosome CIS (Table S5), and 13 CIS after donor chromosome filtering (Table 2). Many of the EL CIS thus fell within the donor chromosomes. We observed a very high insertion rate in the ETS family of transcription factors (TFs), with insertions in *Erg* and *Ets1* in 65% and 44% of ELs, respectively. In fact, only 4 of the 40 ELs lacked insertions targeting either *Erg* or *Ets1* (Table S4). Interestingly, a previous SB insertional mutagenesis study performed in Jak2 mutant mice also identified ETS transcription factors as CIS in EL, and Erg has been implicated in human EL (see discussion), highlighting the importance of ETS TFs in EL^25^. Many CIS occurred near genes implicated in hematologic cancers and hematopoietic development (*Erg, Ets1, Epo, Il2rb, Flt3, Kras, Stat5b, Fli1*). While CIS within donor chromosomes were filtered from our analyses, some of the CIS found within donor chromosomes have been reported in other transposon-mediated insertion studies targeting hematologic neoplasms, and thus may represent physiologically significant gene alterations rather than local hopping. Examples of these types of CIS (on chromosome 4) that are likely to have contributed to EL in our study include *Csf3r* (35% of ELs), *Bach2* (41% of ELs) and *Cdkn2a/Arf* (5% of ELs). Because we found each of these CIS in both T2Onc strains, these insertions were not restricted to potential local hops. TAPDANCE, an alternate statistical method that eliminates donor chromosome insertions, was also used to annotate CIS and gene associations in T-ALL and EL. The most frequent CIS found by TAPDANCE in T-ALL included *Notch1, Ikzf1, Rasgrp1*, *Akt2*, *Runx1, FoxP1*, whereas the most frequent CIS present in EL were *Erg and Ets1*, followed by *Epo, Gata1, Pik3ca* and *Fli1* (Tables S6 and S7).

**Table 2:** Erythroleukemia GCIS (40 tumors total).

Gene	Tumors	Chromosome
*Erg*	27	16
*Ets1*	18	9
*Eras*	8	X
*Il2rb*	4	15
*Dyrk1a*	4	16
*Pop7*	4	5
chr6:31014767–31108367	4	6
*Stat5b*	3	11
*Jarid2*	3	13
*Gigyf1*	3	5
*Flt3*	3	5
*Kras*	3	6
*Fli1*	3	9

### CIS evolution in EL cell lines

**Figure 3:**
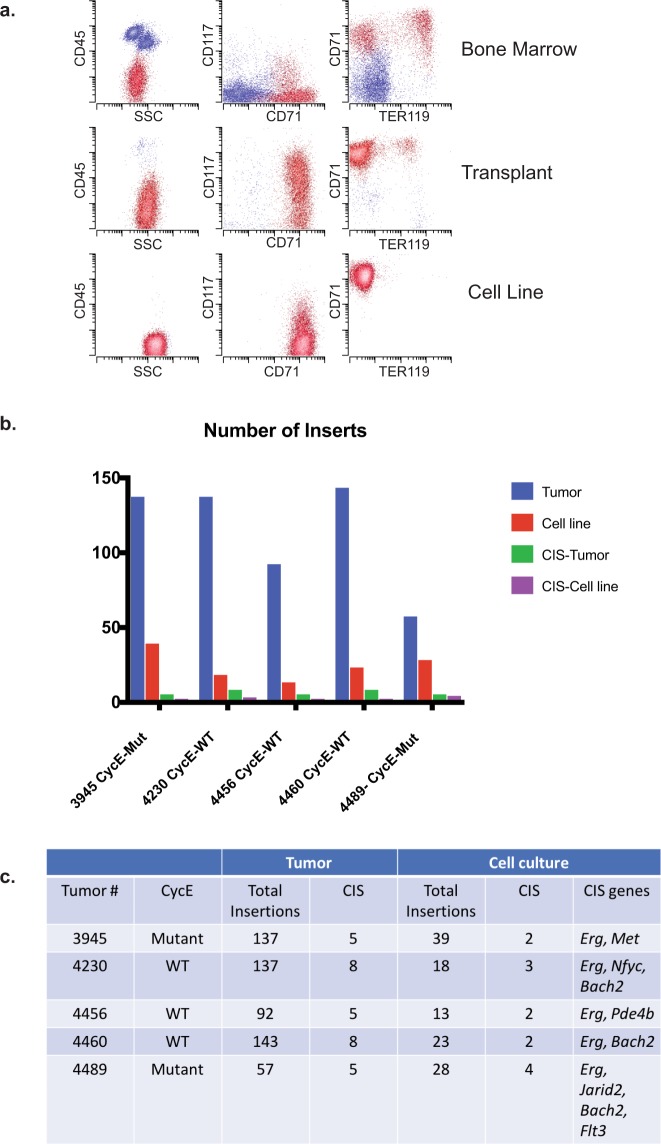
Immunophenotypic change and loss of insertion sites of erythroleukemia following transplantation and cell culture models. (**a**) Flow cytometry results from primary leukemia (top), transplanted leukemia (middle), and cell culture (bottom). The abnormal erythroblasts are CD45 dim/low side scatter/high CD71/variable CD117/variable Ter119 (red). (**b**) Loss of insertion sites following cell culture. Number of total insertion sites and common insertion site (CIS) identified in primary tumor (blue and green) and cell lines (red and purple) in five separate tumors and derived cell lines. (**c**) Quantification of inserts and CIS in tumor-derived cell lines.

We used marrow transplantation to demonstrate that ELs were truly neoplastic and capable of self-replication. Five primary ELs were transplanted into sub-lethally irradiated mice, and recipient mice developed signs of progressive leukemia with a latency of 10–20 days post-injection. Analysis of peripheral blood revealed similar features to the parental tumors, including circulating erythroblasts with the same immunophenotypes as the original tumor, albeit more homogeneously (Fig. 3a). Necropsy of the transplanted mice demonstrated splenomegaly, similar to that seen in the original model. Stable EL cultures were established from five of these transplanted mice using murine erythroblast cell culture media containing c-Kit ligand and erythropoietin. No major immunophenotypic or morphologic changes occurred during the prolonged cell culture (Fig. 3a, Table S2).

We used these long-term EL cultures to further identify putative driver insertions that promote EL. Since the SB transposase is constitutively active in these cell lines, we predicted that serial passage might result in the loss of irrelevant or passenger transposon insertions that were not required for EL proliferation or maintenance in culture, and conversely, that insertions required for tumor maintenance would be preserved. All five EL primary cultures were cultured for 12 weeks in the presence of growth factors and then analyzed for transposon insertions as described above (Table S3). Long-term cell culture dramatically altered the number and identity of the insertions. On average, 113 unique insertions (ranging between 57–143) were identified from the original ELs. Following long-term culture, the number of insertions decreased to an average of 24 unique insertions (ranging from 13–39) per cell line, and only a few of these (1–4) had been previously identified as CIS (Fig. 3b,c). The loss of insertions may result from continued transposon hopping and tumor evolution in culture, or may alternatively reflect clonal selection from an initially heterogeneous population of cells in primary ELs. In all cases, just a few previously identified CIS were maintained during long-term culture (Fig. 3b,c and Table S8). In one cell line, we detected an *Erg* insertion that had not been found in the primary tumor, which may reflect either a small subclonal population present in the original tumor that was not detected in the initial sequencing, or alternatively, clonal evolution *in vitro*. Of the previously identified CIS, only *Erg* insertions were retained in all the cell lines tested, suggesting that persistent *Erg* deregulation is required for EL proliferation and/or maintenance in culture. Interestingly, *Bach2*, a chromosome 4 insertion site that was found in both T2Onc strains, was identified as a CIS in 3/5 cell lines, suggesting that this hematopoietic TF may have an important role in EL^24,26^.

### Effects of Erg insertions and cyclin ET74AT393A expression on EL differentiation and growth factor requirements

**Figure 4:**
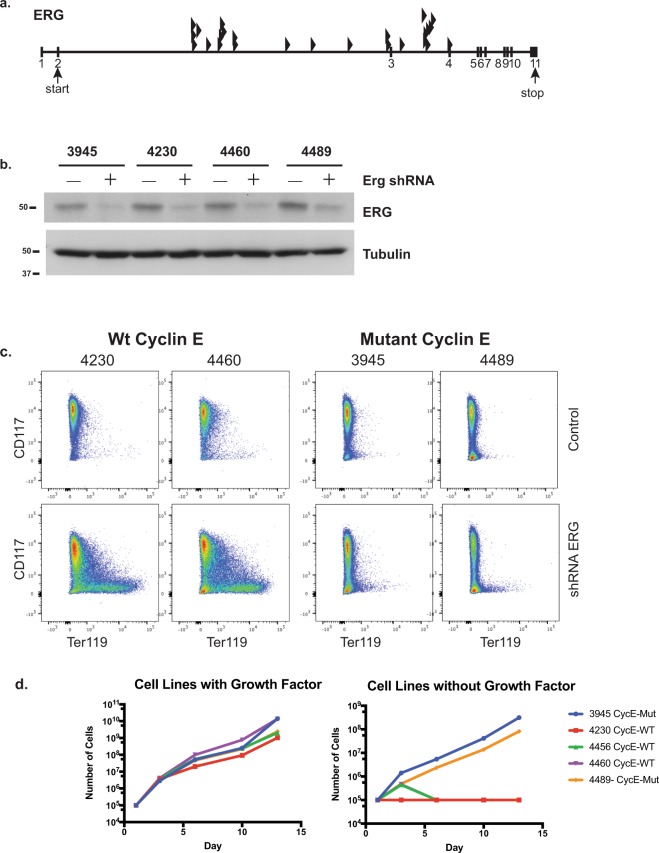
Erg over-expression inhibits erythroid differentiation. (**a**) Transposon insertion sites within *Erg* are predicted to promote the expression of a truncated transcript containing the full ETS domain. All of the insertions are in the same transcriptional orientation as the *Erg* gene. (**b**) Immunoblot showing partial knockdown of ERG with mErg-specific shRNA in EL cell lines. (**c**) ERG knockdown in erythroleukemia cell lines induces immunophenotypic maturation (decreased CD117 and increased Ter119 expression) in wt EL cell lines (4230 and 4460) but not in cyclin ET74AT393A EL cell lines (3945 and 4489). (**d**) Growth factor dependence of the erythroleukemia-derived cell lines. Two cell lines (3945 and 4489) from cyclin ET74AT393A mice are growth factor independent, and three cell lines (4230, 4456, 4460) from wt mice are growth factor dependent (EPO and c-Kit ligand).

*Erg* was the most common EL CIS in our study, with 65% of tumors containing at least one Erg insertion. These integrations were found in the same transcriptional orientation as the ERG gene and upstream of exons 3 and 4, suggesting that the insertions led to overexpression of truncated Erg mRNAs and proteins (Fig. 4a). Erg overexpression drives murine erythroid-megakaryocytic leukemia and has been associated with various human leukemias and poor outcomes^27–29^. We used shRNA to reduce ERG protein expression to study the role of persistent *Erg* deregulation in long-term EL cultures. Primary EL cell lines expressed variable levels of ERG protein, and Erg shRNA only partially reduced ERG abundance (Fig. 4b). The inability to fully silence Erg expression may reflect the essential role of *Erg* deregulation in these cells. Partial *Erg* knockdown induced immunophenotypic differentiation in the cell lines derived from the WT background, with decreased CD117 (cKit) expression and increased Ter119 (glycophorin B), consistent with erythroid maturation (Fig. 4b,c). These results are consistent with prior studies that have shown that ERG expression in human and murine leukemias inhibits differentiation^30–32^. Surprisingly, unlike WT EL cell lines, EL lines derived from cyclin ET74AT393A mice did not exhibit immunophenotypic markers of differentiation after partial ERG knockdown (Fig. 4c). Even more unexpectedly, both cell lines derived from the cyclin E mutant mice proliferated without growth factor supplementation, while the three cell lines derived from wild-type mice required erythropoietin and c-Kit supplementation (Fig. 4d). These results suggest that inappropriate cyclin E regulation confers growth factor independence. This is reminiscent of observations with the related cell cycle protein, cyclin D2, which promotes growth factor independence in B-cell lines^33^. Thus, despite the similar disease spectrum in both strains, cyclin ET74AT393A expression overcame normal growth factor dependency, indicating that cyclin E may be a key mediator of mitogenic signaling in EL cells.

**Figure 5:**
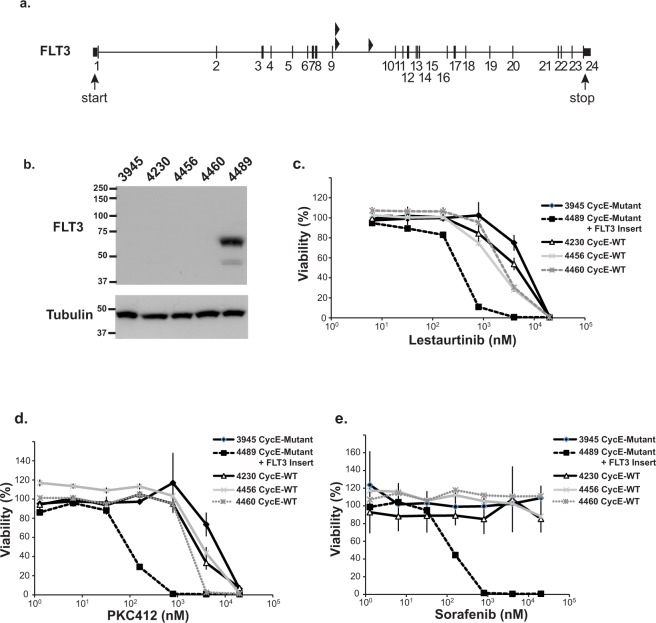
(**a**) Transposon insertion site within the gene encoding Flt3. All of the insertions are in the same orientation of the gene and are predicted to result in overexpression of a truncated protein containing the tyrosine kinase domain of Flt3. (**b**) Immunoblot showing over-expression of truncated FLT3 protein (65 kDa) in the 4489 cell line that contained an insertion in the Flt3 gene. Endogenous FLT3 was not detected (113 KDa). (**c**–**e**) Over-expression of the truncated FLT3 in 4489 imparts increased sensitivity to a panel of FLT3 tyrosine kinase inhibitors including Lestaurtinib (**c**), PKC412 (**d**), and Sorafenib (**e**).

*Flt3* (FMS-like tyrosine kinase 3) is a receptor tyrosine kinase that is mutated in AML and confers a poor clinical prognosis^34^. We identified three *Flt3* insertions in ELs, all of which were located downstream of exon 9 (Fig. 5a). These insertions are predicted to result in overexpression of a truncated FLT3 protein containing only the tyrosine kinase domain without the receptor domain. One of the tumors selected for culture (4489) had the *Flt3* insertion and retained this insertion after serial passage. Western blot analysis shows that this cell line over-expressed a truncated ~65 kDa form of FLT3 protein without apparent expression of endogenous full length Flt3 (Fig. 5b). To test if the *Flt3* insertion is required for proliferation of this EL line, we treated all five of the tumor-derived cell lines with three different pharmacologic inhibitors that target the FLT3 kinase and have been used in clinical trials of patients with FLT3 mutant AML (Lestaurtinib, PKC412, and Sorafenib). Compared with the other cell lines, line 4489 (with the Flt3 insertion) was exquisitely sensitive to all three inhibitors, showing that this EL cell line remained dependent on FLT3 activity for proliferation (Fig. 5c–e).

## Discussion

Based on our previous work showing that mice with a stabilized cyclin E protein develop proliferative and differentiation anomalies in hematopoietic cells, we undertook this study to use SB to identify putative cyclin E-cooperating oncogenes. In light of the phenotypic consequences of cyclin ET74AT393A expression in mice, as well as the roles for cyclin E in human cancer and murine cancer models^7,14,35–37^, it was surprising to find that the cyclin ET74AT393A allele did not alter the incidence, latency, or spectrum of hematologic cancers, or the gene activations observed. The reasons why cyclin ET74AT393A failed to contribute to multistep tumorigenesis in this model remain unclear. The T74AT393A mutation only stabilizes cyclin E in catalytically active complexes, and because many cyclin E-CDK2 complexes are inactive, this mutation has a greater impact on cyclin E-CDK2 activity than cyclin E abundance. In contrast, cyclin E amplifications in human tumors elevate cyclin E abundance, which may or may not increase cyclin E activity (due to compensatory mechanisms that suppress CDK2 activity). Because cyclin E has non-catalytic activities (e.g. replication origin licensing), one possibility is that mutations that differentially impact cyclin E abundance versus activity may lead to different biologic outcomes^38^. However, we do not have evidence to directly support this speculation from this study.

While cyclin ET74AT393A expression did not alter carcinogenesis, it did impact growth factor requirements and differentiation of EL cell lines. Cyclin E-CDK2 activation has previously been proposed to be a crucial feature of the G1 restriction point, which is defined at the point in the G1 phase of the cell cycle after which cells no longer require extracellular mitogens for S-phase entry^39,40^. Our findings that cyclin ET74AT393A rendered two EL cell lines growth factor independent supports this notion, although these studies involved a limited number of cell lines. Of note, our attempts to convert WT-EL cell lines to GF independence through ectopic cyclin ET74AT393A expression were unsuccessful because ectopic cyclin ET74AT393A expression was toxic.

SB activation by Mx-Cre caused two distinct hematologic neoplasms, T-ALL and EL, that differed morphologically, immunophenotypically, and with respect to the identified CIS associated with each disease. While T-ALL has been extensively described in SB models^22^, EL is much less common^25^. In humans, acute erythroleukemia (EL) is a rare and relatively poorly characterized form of acute myeloid leukemia^19^. Until recently, the diagnostic criteria for EL encompassed a diverse set of hematopoietic malignancies that share some degree of erythroid hyperplasia, ranging from an expanded abnormal myeloid blast population with erythroid hyperplasia to a pure erythroid leukemia with erythroid blasts encompassing greater than 80% of the marrow cellularity (2008 WHO; see below). The prior diagnostic criteria for acute erythroleukemia thus included both erythroleukemia (erythroid/myeloid) and pure erythroid leukemia, which occurs less commonly. Because erythroleukemia (erythroid/myeloid) frequently arises from pre-existing MDS (and shares many clinical features of MDS), the WHO recently modified the diagnostic criteria for EL: the more common erythroleukemia (erythroid/myeloid) is now classified as MDS or AML based on the percentage of myeloid blast population, irrespective of the erythroid component^41,42^. Therefore, many cases that had been previously diagnosed as EL are now reclassified as low grade MDS. Pure erythroid leukemia (PEL) remains a distinct diagnostic entity per WHO criteria and is characterized by malignant immature erythroid cells that represent greater than 80% of the marrow. Because of its rare occurrence and previously imprecise diagnostic criteria, our understanding of PEL remains limited. A few small-scale clinical studies on PEL have demonstrated that the leukemic cells usually have complex chromosomal abnormalities, frequent p53 mutations, and extremely poor overall survival^43,44^.

The murine EL that developed in this study most resembled human PEL, with an expanded erythroid blast population (>80% of the marrow cellularity) associated with peripheral cytopenia, circulating erythroid blasts and frequent extramedullary involvement of the liver and spleen. We identified many insertion sites in ELs that target genes involved in myeloproliferative disorders, growth factor signaling, and signal transduction. In accordance with previous work on murine and human EL^27–29^, our studies support a crucial role for ETS transcription factors in the development and maintenance of EL. Most of the ELs that we studied (90%) had inserts in one of the ETS transcription factors (ERG, ETS, or FLI1) and it is tempting to speculate that the few ELs without these insertions may have activated these pathways through other mechanisms. However, we do not have direct evidence to support this speculation. Strikingly, ERG-associated SB insertions were retained in each of the five EL cell lines that we derived from primary ELs, while the vast majority of other CIS found in the primary tumors were not (Fig. 3c). This supports the idea that EL cell lines depend upon continued ERG deregulation, which is consistent with the differentiation caused by partial ERG knockdown (Fig. 4c). Our studies also suggest that Erg may be a good therapeutic target for several forms of AML, including PEL.

The other CIS that was retained in multiple EL cell lines was *Bach2 (*B-lymphoid transcription factor, BTB and CNC Homology 1 Basic Leucine Zipper Transcription Factor 2), a transcriptional repressor that regulates antibody class switching and has been implicated in B-cell neoplasm^45,46^. Bach1 and Bach2 are highly regulated and homologous proteins with key functions in iron hemostasis, erythropoiesis, megakaryopoiesis, and autoimmunity^47^. Our finding that Bach2 insertions were retained in EL cell lines suggests a biologic selection for persistent Bach2 deregulation and perhaps a role for Bach2 in EL development and/or maintenance. During erythropoiesis, elevated levels of free heme bind to and inhibit Bach1, thereby activating the expression of Beta-globin and other genes involved in erythroid differentiation^48^. Although Bach2 functions as a transcriptional repressor during B-cell maturation and has been implicated in B-cell neoplasms, other studies have shown the Bach2 is also regulated by heme binding and that overexpression of Bach2 in hematopoietic stem cells promotes erythroid commitment, suggesting it may also play an early role in directing erythroid lineage fate decisions^49^.

Finally, pure erythroid leukemia is an aggressive poorly characterized form of AML. Now that the WHO has redefined PEL as a distinct form of AML, new clinical studies are needed to identify the molecular pathways that promote PEL. The functional studies presented here provide candidate genes and pathways that should serve as a resource for future studies of human PEL and help define altered pathways that promote this rare, but highly aggressive, leukemia.

## Materials and Methods

### Mice

All mice used in these studies were previously described. Homozygous cyclin ET74AT393A mice were developed and maintained as described^13^. The Cre inducible RosaSBase-LSL transposase and the T2/Onc2 transposon (Lines 6113 and 6070) mice were obtained from N. Copeland^17,18^. Mx1-Cre transgenic mice were obtained from Jackson Laboratories (Bar Harbor, ME). The breeding schemes to generate study mice are shown in Fig. 1a. For Mx1-Cre induction, mice received intraperitoneal injections of polyinosinic: polycytidylic acid (pIpC) (10 μg/g body weight) every other day for a total of five injections. For transplant studies, erythroleukemia cells were expanded *in vitro* prior to injection. Recipient C57/Bl6 mice were sublethally irradiated (700 Gy) 24 hours prior to transplantation and infused with 1 × 10^6^ cultured erythroleukemia cells. Each cell line was transplanted into five recipient mice. Mice were monitored daily using institutional standard procedures and euthanized when indicated. Animal studies and all animal procedures were approved by the Institutional Animal Care and Use Committee (IACUC) and were carried out at the Fred Hutchinson Cancer Research Center (FHCRC). All methods were performed in accordance with the guidelines and regulation established by FHCRC IACUC, Institutional Review, and Institutional Biosafety Committees.

### Histopathological and Hematological Analysis

Mice were euthanized via carbon dioxide inhalation and necropsies were performed. All tissues were harvested and fixed in 10% formalin for 5–7 days and stored in 70% ethanol until submitted for pathological evaluation. Sternums for bone marrow analysis were decalcified in Formical-4 (Decal Chemical Corp) for 24 hrs. after fixation in formalin. After fixation, all samples were submitted to the Experimental Histopathology Core Facility at FHCRC where tissues were processed, embedded in paraffin, cut into slides, and stained with H&E according to standard techniques. Tissues were reviewed by a board-certified hematopathologist (KRL) and a board-certified veterinary pathologist (SEK). Peripheral blood was harvested and analyzed via Siemens Advia 2120i with multispecies software and a manual differential analysis. Lymphomas and leukemias were classified using the Bethesda recommendations for mice^50,51^.

### Flow cytometry

Primary splenocytes and thymocytes were recovered by crushing excised spleen or thymic tissue between glass microscope slides, washing with PBS + 2% FBS, and passing through an 80 µm mesh filter (Sefar, Nitex 03-80/37). Cells were washed once in PBS + 2% FBS and were resuspended in PBS + 2% FBS and kept on ice until analysis. For analysis of bone marrow, marrow was extracted from femurs using a mortar and pestle and processed as above. For analysis of peripheral blood, red blood cells were lysed (155 mM NH_4_CL, 12 mM NaHCO_3_, 0.13 mM EDTA) at room temperature and washed twice in PBS + 2% FBS. Flow cytometry analysis included a 4-color T-cell panel (CD8a-PE, CD4-APC, CD19-APC-Cy7and CD45r/B220-PerCP (BD Biosciences)) and a 6-color erythroid/myeloid panel (CD45-APC-Cy7, CD71-FITC, CD117-PE-Cy7, Ter119-APC, Ly-6G/Ly-6C (Gr1)-PerCP-Cy5.5, and B220-FITC (BD Biosciences) and CD3e Alexa Fluor 488 (BioLegend). Cells were also stained with DAPI to determine cell viability. Flow cytometry was performed on a Canto 2 (Becton Dickinson) or a custom designed LSR2 (Becton Dickinsin, Franklin Lakes, NJ) and analyzed using either FlowJo Software (FlowJo, Ashland, OR) or Woodlist, a noncommercial software program developed in our clinical laboratory^52^.

### Ligation-mediated PCR to amplify transposon junction sequences

Genomic DNA was extracted from frozen tissue using the Puregene Core Kit A (Qiagen) according to manufacturer’s instructions. Five μg of genomic DNA in a volume of 100 μl of ddH2O was sheared to 300 bp lengths using the Covaris E-series sonicator according to the manufacturer’s protocol. One μg of DNA (20 μl) was end-repaired in a reaction containing T4 DNA polymerase and polynucleotide kinase and incubated at 20**°**C for 30 minutes. Then 1 μl of 0.5 M EDTA was added and enzymes were heat-inactivated at 75**°**C for 20 minutes. Blunt/Alu adaptors were ligated to ends of repaired DNA and amplification of transposon junctions was conducted using a ligation-mediated PCR approach as previously described^20,22^. Individual sample library quality was analyzed by running an aliquot via agarose gel electrophoresis. Remaining PCR products were purified, pooled (20–25 ng per sample), and submitted for direct sequencing on an Illumina HiSeq 2000. Sequencing reads were analyzed using gene-centric common insertion site analysis (gCIS) or TAPDANCE^20,21^.

### Cell lines

Primary tumor cell lines were derived by plating splenocytes in IMDM media containing 20% FBS, 4.5 × 10^−5^ M monothioglycerol, 2 U/ml erythropoietin (Epoetin alpha; Amgen Biotechnology), and 50 ng/ml mouse stem cell factor. Cell lines were then maintained in culture as indicated in the text. Lentivirus infection with Erg-specific shRNA, and retroviral infection with cyclin ET74AT393A and wildtype cyclin E were performed as previously described^53^.

### Western Blotting

Western blotting was performed as described^11^. Anti-ERG antibody (CM421AC) was from Biocare Medical (Pacheco, CA) and anti-FLT3 antibody (8F2) was from Cell Signaling (Danvers, MA).

### Cytotoxicity Assays

Cell lines were incubated with the indicated concentrations of drugs and incubated for 72 hours. Viability was measured using a resazurin-based assay and % viability was normalized to the untreated control.

## Supplementary information

Supplemental Information

Table S2

Table S3

Table S4

Table S8
