# Supplementary material for: Insertional mutagenesis using the Sleeping Beauty transposon system identifies drivers of erythroleukemia in mice
Source: Sci Rep. 2019 Apr 2;9:5488. doi: 10.1038/s41598-019-41805-x (PMC6445099; doi:10.1038/s41598-019-41805-x)
Supplement: Supplementary file 1 — Supplemental Information [file 41598_2019_41805_MOESM1_ESM.pdf]

Supplementary Information: Supplemental Figures and legends, 2) list of Supplemental Tables/Datasets.

Insertional mutagenesis using the Sleeping Beauty transposon system identifies drivers of erythroleukemia in mice.

Keith R. Loeb[1,2], Bridget T. Hughes[1,3,4,] Brian M. Fissel[5], Nyka J.Osteen[1], Sue E Knoblaugh[6], Jonathan E. Grim[1.7], Luke J. Drury[8], Aaron Sarver[9], Adam J. Dupuy[8], and Bruce E. Clurman,[1,3],*

Divisions of Clinical Research[1], Public Health Sciences[2] and Human Biology[3], Fred Hutchinson Cancer Research Center, Seattle, WA, 98109, Department of Veterinary Biosciences, College of Veterinary Medicine, The Ohio State University, Columbus, OH 43210[6], VA Puget Sound Health Care System, Seattle, WA, 98108[7], Department of Anatomy & Cell Biology[8], University of Iowa, Iowa City, IA. 52242, Institute for Health Informatics, University of Minnesota, Minneapolis, MN. 55455[9].

Current Addresses

[4]University of Utah, Salt Lake City, UT
[5]Boston University School of Medicine, Boston, MA

*Corresponding Author

Bruce E Clurman, M.D., Ph.D.
Fred Hutchinson Cancer Research Center
Fairview Ave N, D2-100
Seattle, WA. 98109
Tel: 206-667-4525; bclurman@fredhutch.org;

Supplemental Figures and legends

Figure S1. Morphology and immunophenotype of T-cell lymphoblastic lymphomas. Histologic sections (H&E) of normal thymus (A-2.5 objective) and enlarged thymic lymphomas (B-2.5x objective left and 20X objective right panel). The thymic lymphoma has a homogeneous population of cells admixed with tingible body macrophages. Immunophenotype of normal thymus (CD4+/CD8+) with maturation to single positive cells (CD4 or CD8). (C) Immunophenotype of T-cell lymphoma/leukemia (CD8+ cells with variable loss of CD4 expression) in bone marrow spleen, thymus and peripheral blood (D).

Figure S2. Variable immunophenotype of selected erythroid leukemias. Flow cytometry plots of the spleen and peripheral blood from three separate mice (4459, 4317 and 4227) demonstrating immunophenotypic variability of erythroid leukemias (red) indicating varied maturation. All had dim CD45, moderate to bright CD71 with variable expression of CD117 (immature marker) and Ter119 (glycophorin; mature marker).

Figure S3. Preserved erythroleukemia immunophenotype in peripheral blood, bone marrow and spleen. Flow cytometry plots of erythroleukemia from peripheral blood, bone marrow and spleen from same mouse demonstrating systemic disease with preserved immunophenotype. Erythroleukemia is shown in red.

Figure S4. Variable megakaryocytic differentiation of erythroleukemias. Flow cytometry plots showing variable expression of CD41 (megakaryocyte marker) in two separate erythroleukemias (4460 and 4230). Note only a variable subpopulation of the cells demonstrate expression of CD41 (blue).

Supplemental Tables

Supplemental Table S1. Loss of GFP positive cells following MC-Cre inductions.

Supplemental Table S2. List and characterization of all T-All and ELs.

Supplemental Table S3. List of all sequenced transposon insertion sites in primary tumors.

Supplemental Table S4. List of all CIS identified by gCIS in T-ALL.

Supplemental Table S5. List of all CIS identified by gCIS in EL.

Supplemental Table S6. List of all CIS identified by TAPDANCE in T-ALL.

Supplemental Table S7. List of all CIS identified by TAPDANCE in EL.

Supplemental Table S8. List of all sequenced transposon insertion sites in EL cell lines.

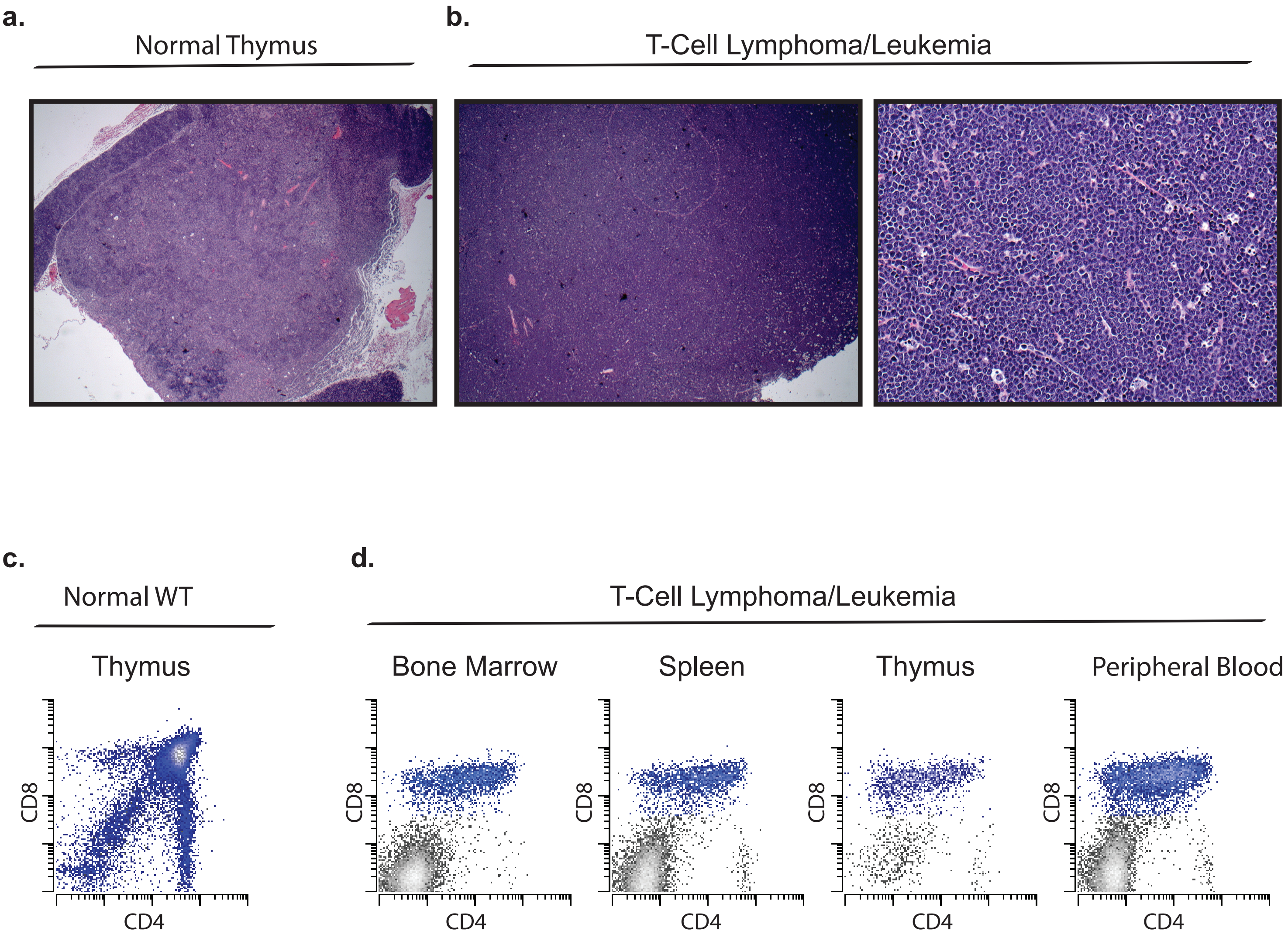
a.
Normal Thymus
b.
T-Cell Lymphoma/Leukemia
c.
Normal WT
Thymus
CD8
CD4
d.
T-Cell Lymphoma/Leukemia
Bone Marrow
Spleen
Thymus
Peripheral Blood

Figure S1

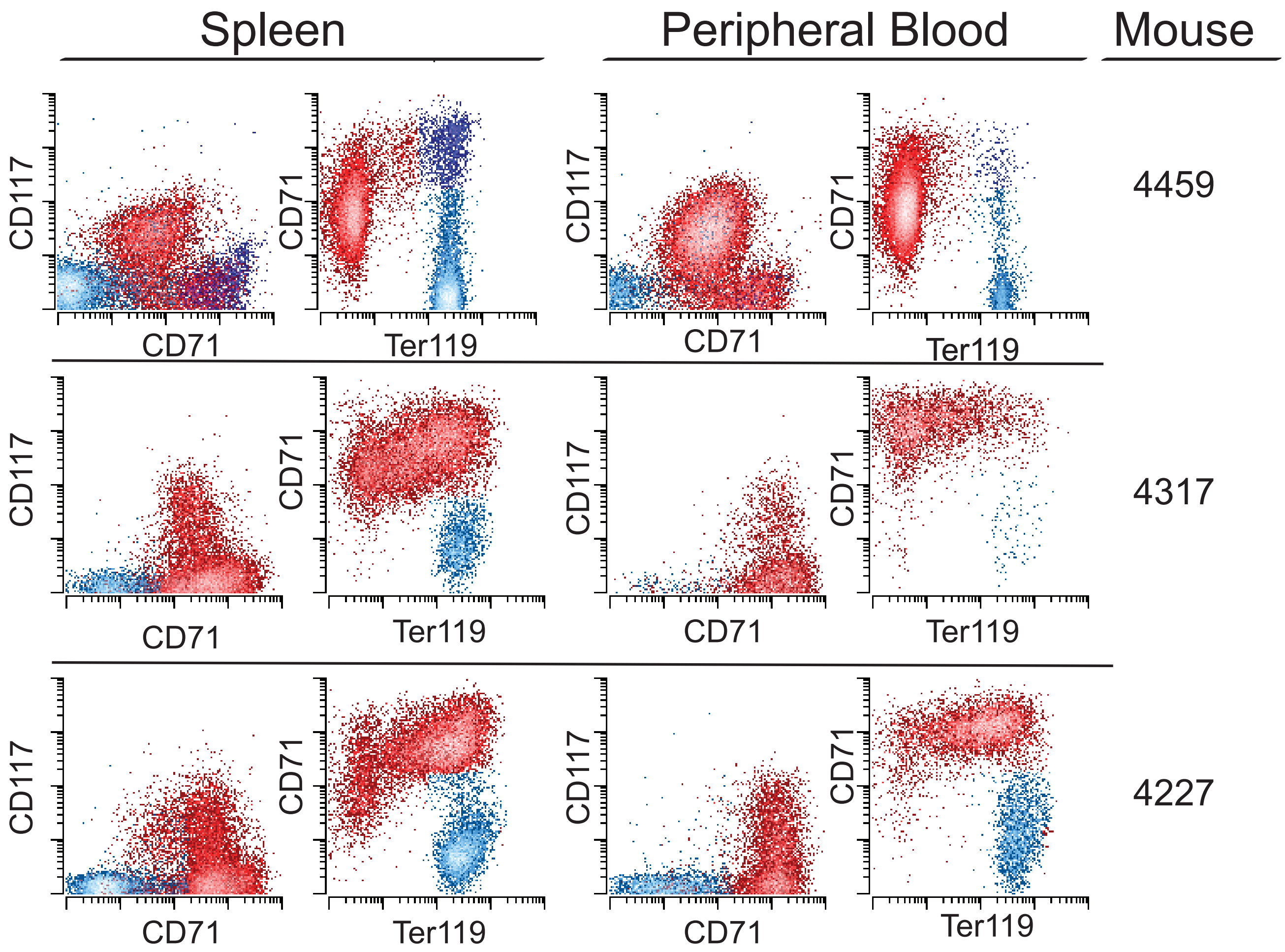
Spleen
Peripheral Blood
Mouse
CD117
CD71
CD71
Ter119
4459
4317
4227

Figure S2

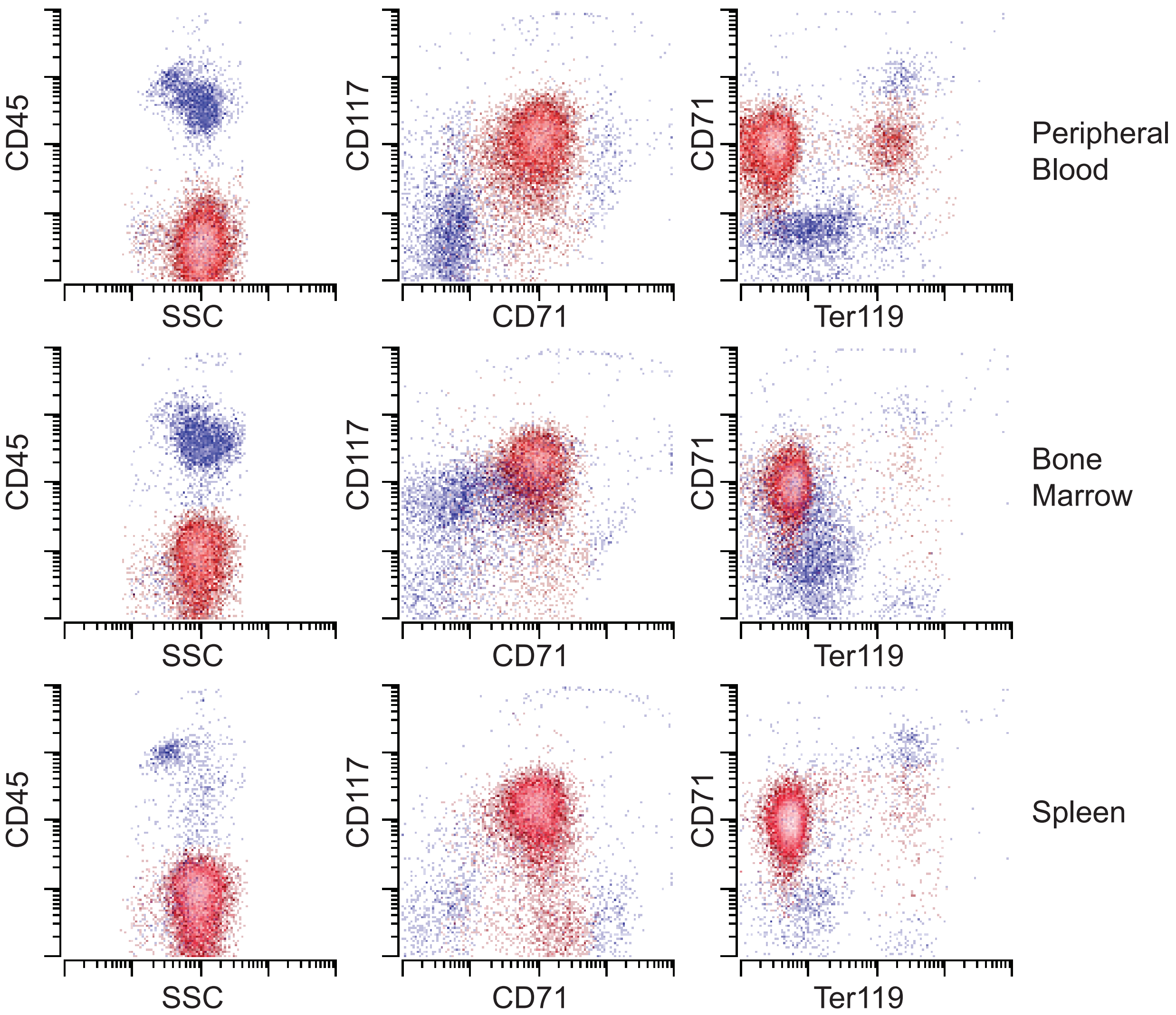
CD45
SSC
CD117
CD71
CD71
Ter119
Peripheral Blood
CD45
SSC
CD117
CD71
CD71
Ter119
Bone Marrow
CD45
SSC
CD117
CD71
CD71
Ter119
Spleen

Figure S3

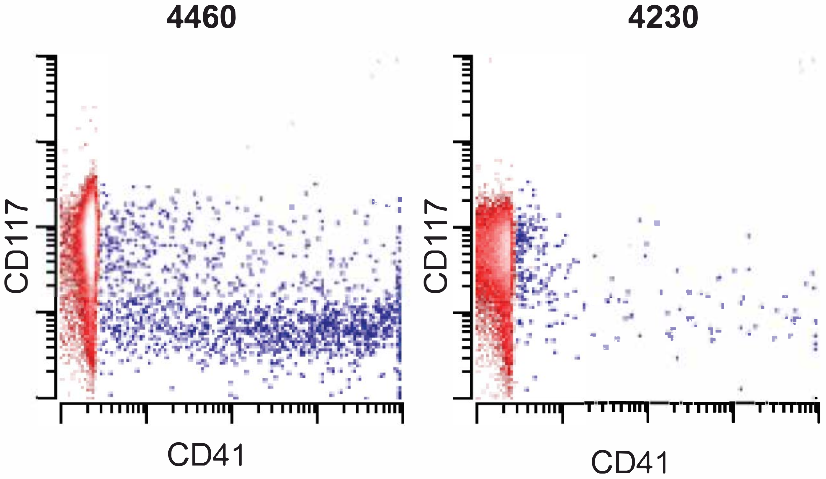

Figure S4

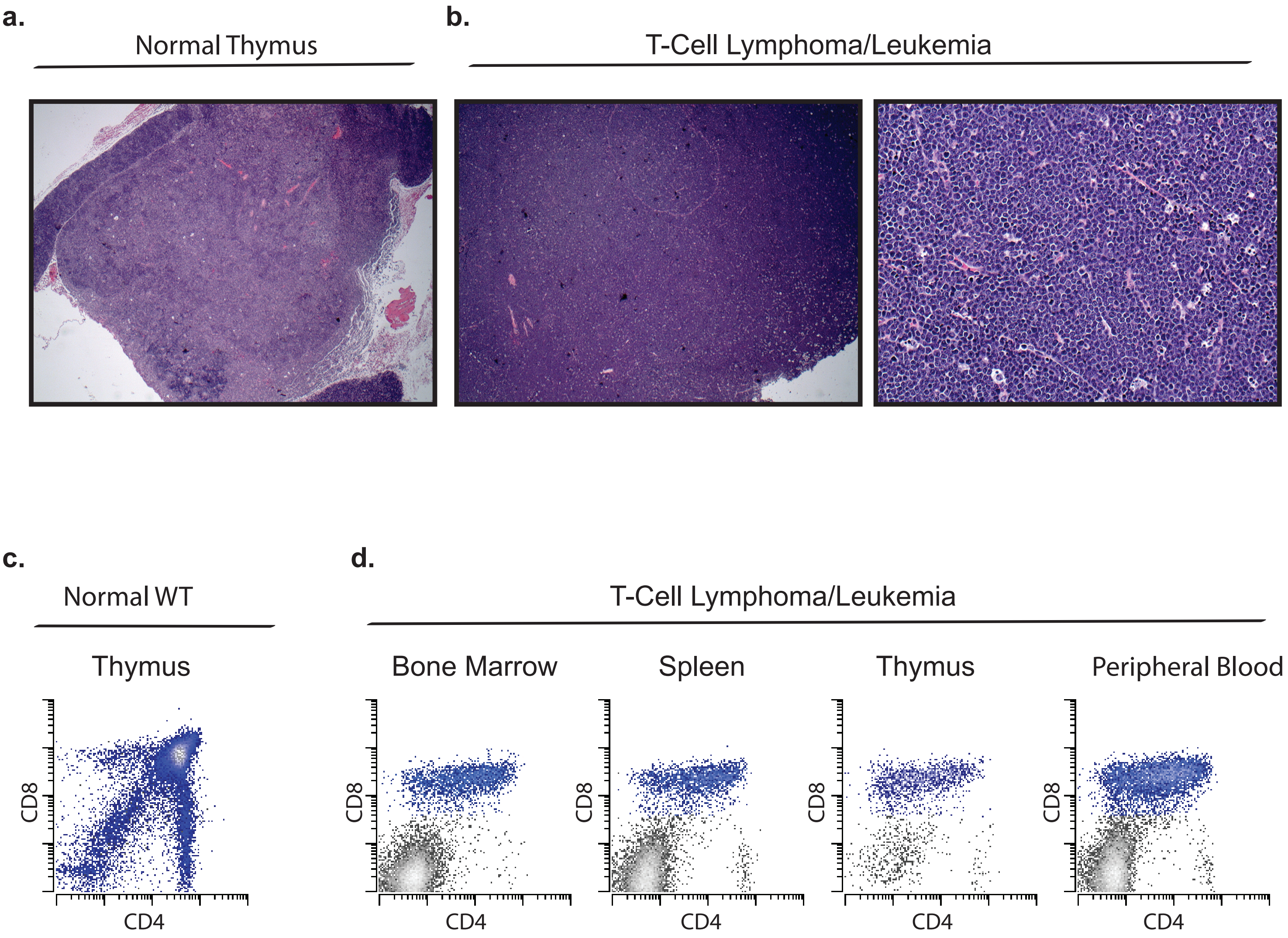
a.
Normal Thymus
b.
T-Cell Lymphoma/Leukemia
c.
Normal WT
Thymus
CD8
CD4
d.
T-Cell Lymphoma/Leukemia
Bone Marrow
Spleen
Thymus
Peripheral Blood
CD8
CD4

Figure S1

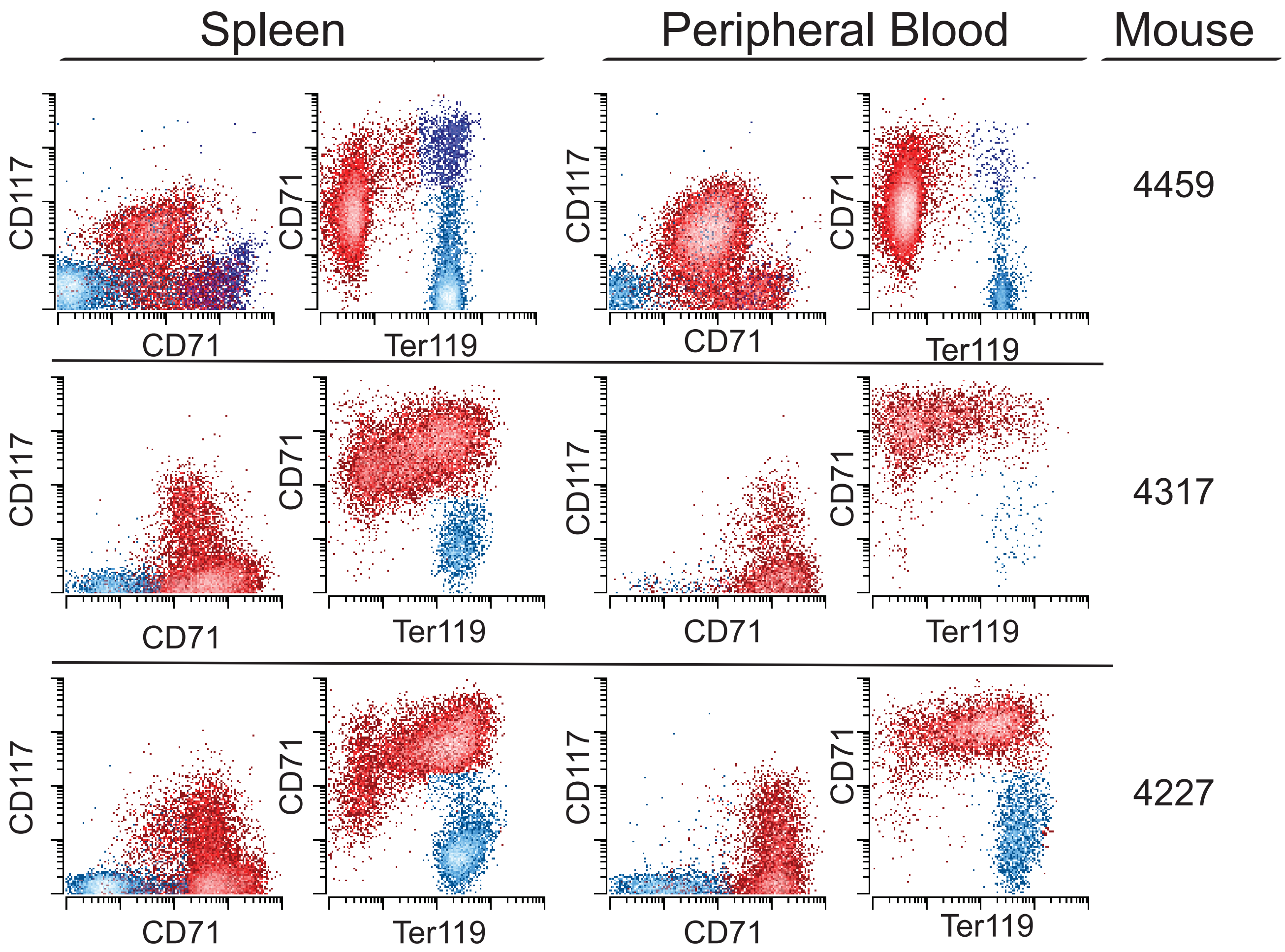
Spleen
Peripheral Blood
Mouse
CD117
CD71
CD71
Ter119
4459
4317
4227

Figure S2

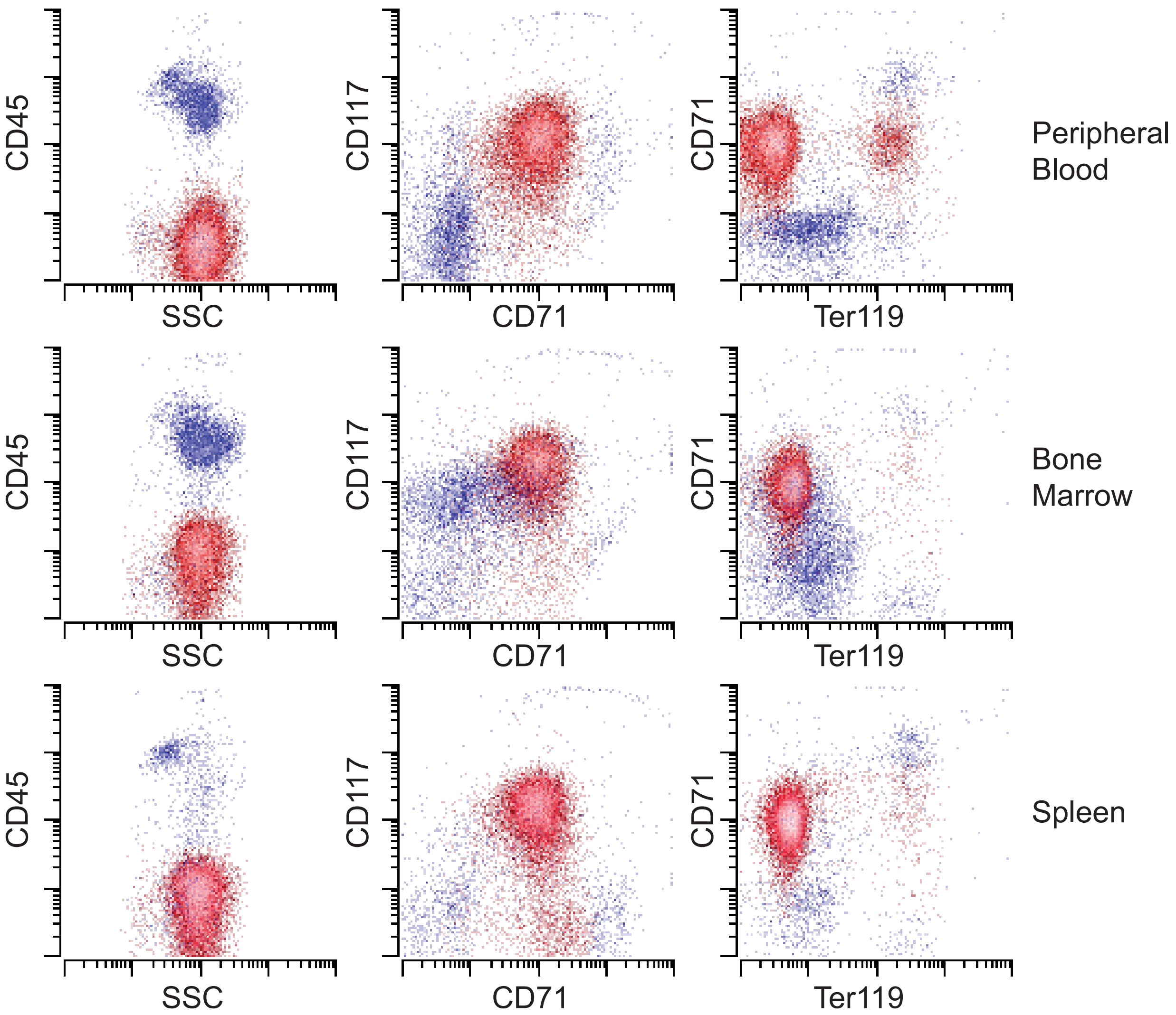
CD45
SSC
CD117
CD71
CD71
Ter119
Peripheral Blood
CD45
SSC
CD117
CD71
CD71
Ter119
Bone Marrow
CD45
SSC
CD117
CD71
CD71
Ter119
Spleen

Figure S3

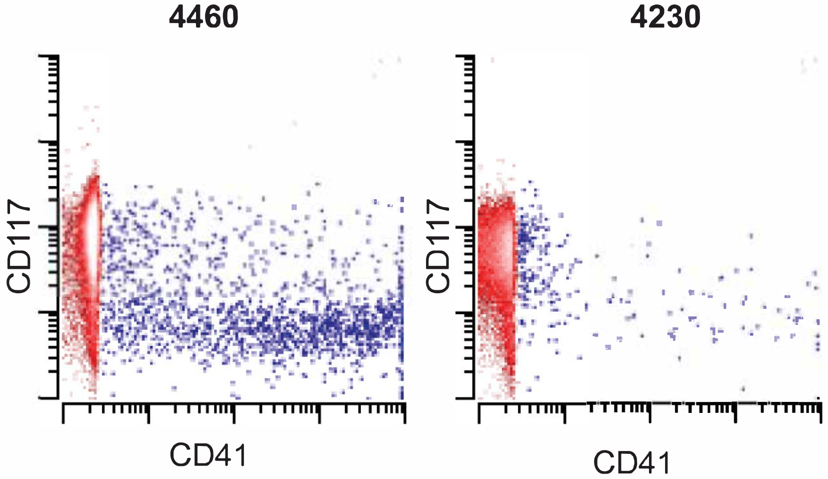

Figure S4

Supplemental Table 1. Monitoring Mx-Cre activation by loss of GFP

Mouse	GFP positive	GFP Negative
**3566 (no Poly IC)**	93.65%	3.97%
**3999**	14.9%	81.3%
**4038**	14%	85.8%
**4064**	20%	79.9%
**4088**	19.5%	80.4%
**3975**	18.5%	81.3%
**4037**	27.3%	72.7%
**4160**	19.5%	80.5%
**4162**	19.9%	80.1%
**3672**	22.50%	76.20%
**3674**	27.65%	71.15%
**3685**	22.40%	76.90%
**3596**	20.25%	79.10%
**3677**	20.35%	79.20%
**3680**	16.35%	81.90%
**3684**	23.65%	75.85%

Supplemental Table 5. Total gCIS identified in Erythroleukemias (40 tumors total)

Gene Symbol	# of tumors	% of Tumors	Chromosome
ERG	22	65	16
ETS1	14	41	9
CSF3R	11	32	4
BACH2	8	24	4
ERAS	6	18	X
CNTNAP5B	5	15	1
FAF1	5	15	4
NFIA	5	15	4
NFYC	5	15	4
PDE4B	5	15	4
BCL2	4	12	1
CDKN2A	4	12	4
DYRK1A	4	12	16
NCOA2	4	12	1
PKHD1	4	12	1
TLE1	4	12	4
**0610043K17RIK**	3	9	4
ACER2	3	9	4
BC057079	3	9	4
CLASP1	3	9	1
FLI1	3	9	9
FLT3	3	9	5
GIGYF1	3	9	5
JARID2	3	9	13
KRAS	3	9	6
MEGF9	3	9	4
MRPS9	3	9	1
MTAP2	3	9	1
MYO1B	3	9	1
PLCL1	3	9	1
POP7	3	9	5
PTPN4	3	9	1
SCMH1	3	9	4
STAT5B	3	9	11
TBC1D8	3	9	1
TRIP12	3	9	1

Supplemental Table 6. Tapdance identification of CIS from T-cell lymphoma/leukemias (53 tumors total)

Gene	Tumors	Chromosomes
Notch1	40	2
Ikzf1	26	11
Erg	23	16
Rasgrp1	19	2
Akt2	14	7
Runx1	10	16
Zmiz1	8	14
Foxp1	7	6
Akt1	6	12
Runx2	5	17
Kras	5	6
Sik3	4	9
Sos1	4	17
Crebbp	4	16
Scai	4	2
Thsd7a	3	6
Adamts10	3	17
Ptger4	3	15
Nedd9	3	13
Pcdh9	3	14
Fntb	3	12
Stat5a	3	11

Supplemental Table 7. CIS identified by Tapdance from Erythroleukemia (40 tumors total)

Gene	# Tumors	Chromosome
Erg	21	16
Ets1	15	9
Epo	6	5
Gata1	6	X
Pik3ca	3	3
Fli1	4	9
RP23-459L15.1	5	6
AC116499.1	5	8
Dyrk1a	4	16
Flt3	4	5
Ppargc1a	3	5
Gm9954	3	5
Stat5a	3	11
Cbl	3	9
